# VIASCKDE Index: A Novel Internal Cluster Validity Index for Arbitrary-Shaped Clusters Based on the Kernel Density Estimation

**DOI:** 10.1155/2022/4059302

**Published:** 2022-06-08

**Authors:** Ali Şenol

**Affiliations:** Department of Computer Engineering, Faculty of Engineering, Tarsus University, Mersin, Turkey

## Abstract

The cluster evaluation process is of great importance in areas of machine learning and data mining. Evaluating the clustering quality of clusters shows how much any proposed approach or algorithm is competent. Nevertheless, evaluating the quality of any cluster is still an issue. Although many cluster validity indices have been proposed, there is a need for new approaches that can measure the clustering quality more accurately because most of the existing approaches measure the cluster quality correctly when the shape of the cluster is spherical. However, very few clusters in the real world are spherical. Therefore, a new Validity Index for Arbitrary-Shaped Clusters based on the kernel density estimation (the VIASCKDE Index) to overcome the mentioned issue was proposed in the study. In the VIASCKDE Index, we used separation and compactness of each data to support arbitrary-shaped clusters and utilized the kernel density estimation (KDE) to give more weight to the denser areas in the clusters to support cluster compactness. To evaluate the performance of our approach, we compared it to the state-of-the-art cluster validity indices. Experimental results have demonstrated that the VIASCKDE Index outperforms the compared indices.

## 1. Introduction

Clustering approaches are unsupervised learning techniques that separate data into groups called clusters according to the similarities and dissimilarities among the data [1, 2]. The DBSCAN [3], kmeans [4], BIRCH [5], Spectral Clustering [6], Agglomerative Clustering [7], HDBSCAN [8], Affinity Propagation [9], and OPTICS [10] are some examples of them, and they are used in many fields such as pattern recognition [11–13], machine learning [14–16], data mining [17, 18], web mining [1, 19], bioinformatics [20, 21], and streaming data mining [22, 23]. On the other hand, measuring the performance of any proposed clustering approach is also an important issue because each algorithm has its special point of view, and the results of each clustering technique vary. Therefore, to overcome this problem, cluster validation analysis or cluster validation indices have emerged. These approaches are generally used for two purposes, which are measuring the performance of clustering algorithms and contributing to clustering algorithms as a guide by finding the optimum number of clusters.

Cluster validation indices are divided into two main categories as internal and external indices. In external indices, true class labels are compared with the labels that are assigned by the proposed algorithm to measure the performance. Therefore, to use these indices, there is a need for true class labels. The Purity [24], Rand Index [25], Adjusted Rand Index [26], Accuracy, Precision and Recall [27], F-Measure [28], and NMI [29] can be given as examples of these types of indices. On the other hand, in the internal indices, we do not need actual class labels to measure the quality of clusters. In these indices, the evaluation of clustering performance is based on how similar the data in the same cluster are to each other, known as compactness, and how dissimilar the data in different clusters are from each other, known as separation. The Silhouette Index (SI) [30], Dunn Index [31], Davies–Bouldin (DB) [32], Calinski-Harabasz (CH) [33], Xie-Beni (XB) [34], S_Dbw [35], and RMSSTD [36] can be mentioned as primary cluster validity indices. Besides, there are many new cluster validity indices such as the CVNN [37], CVDD [38], DSI [39], SCV [40], and AWCD [41].

The main problem of the majority of state-of-the-art cluster validity indices is that they measure the cluster quality correctly when the shapes of the clusters are spherical. As an example, Silhouette Index (SI) uses the means of distances of each data in the cluster to evaluate their quality. Similarly, Davies–Bouldin (DB) uses cluster diameters and cluster centroids, and the Calinski-Harabasz (CH) uses the square of intracluster and intercluster distances. These all calculations are ideal if the shape of the cluster is spherical. However, the shapes of the minority of clusters are spherical in the real world. Additionally, if the shape is arbitrary, these indices cannot measure the cluster quality correctly because the center of gravity of any cluster is in the middle only if the shape is spherical.

Similar to our approach, there is another kernel density estimation-based cluster validation index, named the *M*_*clus*_ [42]. In the *M*_*clus*_, the authors used a function of estimation of the mode to assess cluster quality. This mode function allows the index to assess the cluster quality by adopting interpoint distance measures that can be defined to have a probability density function. To evaluate clustering with the number of clusters greater than 1 (*K* > 1), they applied the mode estimation procedure for interpoint distances that are assumed to have a probability density function between the data members. On the other hand, in this study, we proposed a novel Internal Validity Index for Arbitrary-Shaped Clusters based on the kernel density estimation (the VIASCKDE Index). We aimed to calculate the cluster quality accurately by using compactness and separation of each data to support arbitrary-shaped clusters and the kernel density estimation (KDE) to weight denser regions in the clusters to the compactness of the clusters. Therefore, the advantages of our new approach can be listed as follows:

- The VIASCKDE Index can evaluate arbitrary-shaped clusters correctly
- It weights denser regions to support the compactness of clusters
- It is suitable for all types of clustering techniques, especially for density-based algorithms
- It can be used for micro-cluster-based approaches
- It has greater performance when compared with state-of-the-art techniques

The rest of this paper was organized as follows: in Section 2, the related studies were reviewed. In the 3^rd^ section, the problem with existing works and the need for the proposed approach was explained. While details about the VIASCKDE Index were given in the 4^th^ section, the comparison of experimental results with the state-of-the-art approaches on real and synthetic datasets was given in the 5^th^ section. After that, the discussion on the results was provided in Section 6. Finally, the conclusion of the study was presented in Section 7.

## 2. Background and Related Works

As cluster validation techniques, in internal methods, we do not need the actual class labels. The cluster validation operation is done by calculating the similarities in the intraclusters and the differences in the interclusters produced by the model to reveal how consistent the produced clusters are [43]. As mentioned above, in the internal methods, cluster quality is evaluated in the aspects of two concepts [44]:

1. *Compactness*: it states how much the data, which is in the same cluster, are close to each other. Closer data mean better clustering.
2. *Separation*: it evaluates how much the clusters are far from each other. In the clustering evaluation, it is expected to be far from each other as much as possible.

**Figure 1:**
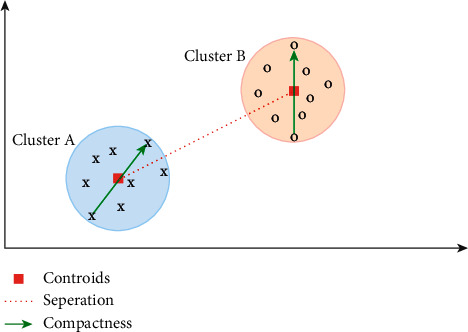
The example of the relationship between the compactness and separation concepts of two clusters in a two-dimensional data space.

The illustration of these two concepts is presented in Figure 1, while the equation is demonstrated in Eq. (1). Here, *α* and *β* are the weights.

(1) $$\begin{array}{c} \text{Index}=\frac{\alpha •\text{Compactness}}{\beta •\text{Separation}}. \end{array}$$

**Figure 2:**
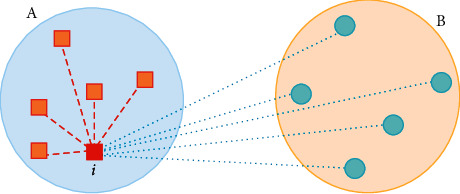
The example of Silhouette Index.

There are many internal methods proposed in the literature. In this section, we focused on the validation indices that are relevant to our approach. To make definitions shorter and more understandable, the general definitions are as follows:

Let *X* *=* *{x*_*1*_*, x*_*2*_*,…,x*_*n*_*} ∈ R*^*d*^ be a dataset containing *n* points in a *d*-dimensional space, and *x*_*i*_ *∈* *R*^*d*^. *X* is a set of disjoint *k* clusters (where *C*_*i*_ is a cluster and *i* *=* *1,2,3,…,k*), and *n*_*i*_ data are in the *C*_*i*_ cluster. While the cluster center that is the gravity center of cluster *C*_*i*_ is the mean of the data that belongs to *C*_*i*_ and calculated by *μ*=1/*n*_*i*_∑_*x*_*i*_∈*C*_*i*__*x*_*i*_, the mean of all datasets is calculated by $\bar{\mu }=1/n\sum_{x\in X}x$. In the present study, the mentioned distance is the Euclidean distance; one of each *x* and *y* is data of the dataset, and the Euclidean distance between these two data is expressed as *d*_*e*_*( x *, *y *). In light of this information, we can briefly list the main internal cluster validity indices as follows:

*Silhouette Index (SI)* [30]: as given in Figure 2, the compactness value of one of the data in any cluster is calculated by measuring the distance from the data to each data in the same cluster. Then, the compactness of the cluster, which is notated as *a(x)*, is calculated by measuring the mean of compactness of all the data that the cluster has. The average of the distances from the elements of the nearest cluster, to which the mentioned data do not belong, gives the separation value of that data. After that, the separation value of the cluster is found by calculating the mean of the separation values of all the data of the cluster and it is notated as *b(x)*. From now on, we can calculate the SI value, which is the cluster validity index of the model. The equations to calculate *SI*, *a(x),* and *b(x)* are given in equations (2)–(4), respectively. The SI value is [−1, +1]. While -1 means the worst clustering, +1 means the best clustering.

(2) $$\begin{array}{c} a\left(x\right)=\frac{1}{n_{i}-1}\underset{x,y\in C_{i},y\neq x}{\sum }\;d_{e}\left(x,y\right), \end{array}$$

(3) $$\begin{array}{c} b\left(x\right)={\text{min}}_{j=1,2,\ldots ,k;j\neq i}\left\{\frac{1}{n_{j}}\underset{x\in C_{j},y\in C_{j}}{\sum }\;d_{e}\left(x,y\right)\right\}, \end{array}$$

(4) $$\begin{array}{c} SI=\frac{1}{n}\overset{k}{\underset{i=1}{\sum }}\underset{x\in C_{i}}{\sum }\frac{b\left(x\right)-a\left(x\right)}{\text{max}\left(a\left(x\right),b\left(x\right)\right)}. \end{array}$$

*Dunn Index (DI)* [31]: the DI calculates the success of the model based on compactness and the separation between the clusters. To do this, the DI value of a cluster is calculated by the distance to the closest cluster and its own diameter. Let *d*_min_(*C*_*i*_, *C*_*j*_) be the closest distance between clusters *C*_*i*_ and *C*_*j*_, and let *diam(C*_*l*_) be the diameter of the cluster *C*_*l*_, and the values of these two variables are calculated by *d*_*min*_(*C*_*i*_, *C*_*j*_)=*min*_*x*_*i*_∈*C*_*i*_, *x*_*j*_∈*C*_*j*__*d*_*e*_(*x*_*i*_, *y*_*i*_)  and *diam*(*C*_*l*_)=*max*_*x*_*i*_∈*C*_*i*_, *x*_*j*_∈*C*_*j*__*d*_*e*_(*x*_*i*_, *y*_*i*_). Therefore, by knowing the value of *d*_min_(*C*_*i*_, *C*_*j*_) and *diam(C*_*l*_), the *DI* of the model is calculated by equation (5). The larger the result value, the more successful the clustering is.

(5) $$\begin{array}{c} DI={\text{min}}_{1\leq i\leq k}\left\{{\text{min}}_{j\neq i,\;1\leq j\leq k}\left\{\frac{\text{dmin}\left(C_{i},\;C_{j}\right)}{{\text{max}}_{1\leq l\leq k}\left\{\text{diam}\left(C_{l}\right)\right\}}\right\}\right\}. \end{array}$$

*Calinski-Harabasz (CH)* [33]: the CH calculates compactness and separation values via the mean of the squares of the interclass and intraclass distances. The CH index value is calculated by (6). In the CH index, the goal is to make the result as large as possible.

(6) $$\begin{array}{c} CH=\frac{\sum_{i=1}^{k}\;n_{i}d_{e}^{2}\left(\mu_{i},\mu \right)/\left(k-1\right)}{\sum_{i=1}^{k}\sum_{x\in C_{i}}\;d_{e}^{2}\left(x,\mu_{i}\right)/\left(n-k\right)}. \end{array}$$

*Davies–Bouldin (DB)* [32]: the compactness value is calculated over the mean of the variance of the data in each cluster. On the other hand, the separation value is calculated over the distance from the center of the cluster to the center of the closest one. Let *avg(C*_*i*_), which is calculated by (7), be the average of the distances of each data in the cluster *i* to the cluster center, and the *avg(C*_*i*_) is calculated by (8).

(7) $$\begin{array}{c} avg\left(C_{i}\right)=\frac{1}{n_{i}\left(n_{i}-1\right)}\underset{x_{i},\;x_{j}\in C_{i}}{\sum }d_{e}\left(x_{i},x_{j}\right), \end{array}$$

(8) $$\begin{array}{c} DB=\frac{1}{k}\overset{k}{\underset{i=1,\;i\neq j,\;1\leq j\leq k}{\sum }}\text{max}\left\{\frac{\text{avg}\left(C_{i}\right)+\text{avg}\left(C_{j}\right)}{d_{e}\left(\mu_{i},\mu_{j}\right)}\right\}. \end{array}$$

*S_Dbw Index* [35]: The S_Dbw calculates the compactness value of the clusters over the standard deviations (*σ*) of the data that the cluster has. On the other hand, it calculates the separation value by the distance between the centers of the clusters. The S_Dbw index is a type of index that considers the density of clusters. Let *den* be the density of the cluster, and the S_Dbw index value is calculated with the following equations:

(9) $$\begin{array}{c} S\_Dbw=\frac{1}{k}\underset{C_{i}\in C}{\sum }\frac{\sigma \left(C_{i}\right)}{\sigma \left(X\right)}+\;\frac{1}{k\left(k-1\right)}\underset{C_{i}\in C}{\sum }\underset{C_{j}\in C,C_{i}\neq C_{j}\;}{\sum }\frac{\text{den}\left(C_{i},\;C_{j}\right)}{\text{max}\left\{\text{den}\left(C_{i}\right),\text{den}\left(C_{j}\right)\right\}},\\ \text{den}\left(C_{i}\right)=\underset{x_{p}\in \;C_{i}}{\sum }f\left(x_{p},\mu_{i}\right),\\ \text{den}\left(C_{i},\;C_{j}\right)=\underset{x_{p}\in \;C_{i}\cup^{\;}C_{j}}{\sum }f\left(x_{p},\frac{\mu_{i}+\mu_{j}}{2}\right),\\ f\left(x_{p},\;\mu_{i}\right)=\left\{\begin{array}{cc} 0, & d_{e}\left(x_{p},\;\mu_{i}\right)>\sigma \left(C\right),\\ 1, & \text{otherwise.} \end{array}\right. \end{array}$$

*Distance-based Separability Index* (DSI) [39]: the DSI is another approach that measures the cluster quality by the means of the distances based on intercluster and intracluster. Let *C*_*i*_ and *C*_*j*_ be two clusters and have *N*_*i*_ and *N*_*j*_ data points, respectively. The intracluster distance set of cluster *C*_*i*_ will be a set as given equation (13). Moreover, the intercluster distance set is measured based on the distances of data pairs of clusters *C*_*i*_ and *C*_*j*_. To compute the DSI, the Kolmogorov–Smirnov (KS) test was utilized.

(10) $$\begin{array}{c} \left\{\begin{array}{c} \left\{d_{C_{i}}\right\}=\left\{\;d_{e}\left(x,\;y\right)|\;x,y\in C_{i};x\neq y\right\},\\ If\;\left|C_{i}\right|=N_{i,},\text{ then }\left|\left\{d_{C_{i}}\right\}\right|=\frac{1}{2}N_{i,}\left(N_{i,}-1\right), \end{array}\right.\\ \left\{\begin{array}{c} \left\{d_{C_{i,j}}\right\}=\left\{\;d_{e}\left(x,y\right)|\;x\in C_{i};y\in C_{j}\right\},\\ If\;\left|C_{i}\right|=N_{i,},\;\left|C_{j}\right|=N_{j,},\text{then }\left|\left\{d_{C_{i,j}}\right\}\right|=N_{i}.N_{j}. \end{array}\right. \end{array}$$

Let *S*_*C*_*i*__ be Kolmogorov–Smirnov test of cluster *C*_*i*_, which is calculated as *S*_*C*_*i*__=*KS*({*d*_*C*_*i*__}, {*d*_*C*_*i*,*j*__}) and *S*_*C*_*j*__ be of *C*_j_, and the DSI of these two clusters is the result of the following equation:

(11) $$\begin{array}{c} \;DSI\left(\left\{C_{i},C_{y}\right\}\right)=\frac{S_{C_{i}}+S_{C_{j}}\;}{2}. \end{array}$$

RMSSTD [35]: the root-mean-square standard deviation (RMSSTD) aims to calculate the clustering quality by measuring the homogeneity of clusters. It is commonly used for hierarchical clustering. Let the dataset consists of *k* clusters, *p* be the number of independent variables, ${\bar{x}}_{ij}$ be the mean of data in variable *j* and cluster *i*, and *n*_*ij*_ is the number of data in variable *p* and cluster *k*. RMSSTD is measured by equation (12). The lower RMSSTD means better clustering.

(12) $$\begin{array}{c} \text{RMSSTD}=\sqrt{\frac{\sum_{i=1,2,\ldots ,p}^{j=1,2,\ldots ,k}\sum_{a=1}^{n_{ij}}{\left(x_{a}-{\bar{x}}_{ij}\right)}^{2}}{\sum_{i=1,2,\ldots ,n}^{j=1,2,\ldots ,k}\left(n_{ij}-1\right)}}. \end{array}$$

## 3. Statement of the Problem

**Figure 3:**
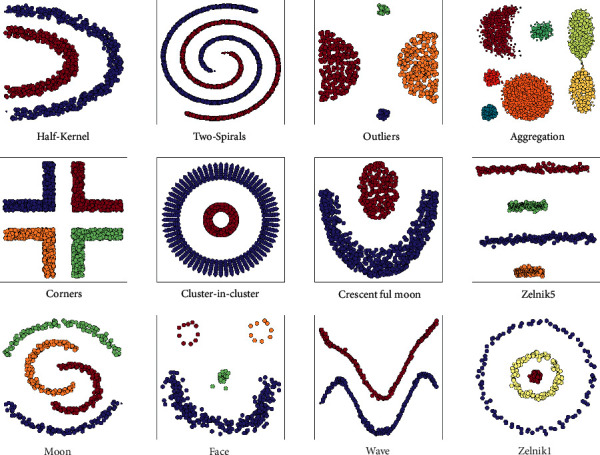
Some examples of the arbitrary-shaped cluster.

Although many approaches have been proposed, analysis of the cluster quality is still an issue. Because there are many clustering approaches in the literature, they differ from each other in many aspects. Therefore, no cluster validation technique can evaluate the quality of all produced clusters precisely. However, some approaches have been used in this task including the Silhouette Index, Dunn Index, Davies–Bouldin, Calinski-Harabasz, and S_Dbw. Although these indices have been used commonly, each of them has a specific problem with cluster validation as given in Table 1. For example, a significant part of the proposed cluster validity indices assumes the shapes of clusters are spherical. In fact, the minority of clusters are spherical in the real world as some examples are given in Figure 3. The SI can be given as an example of these kinds of indices. It cannot achieve a good score if the shape of the cluster is not spherical. On the other hand, the DB and the CH identify clusters that are compact and well separated. However, in the real world, very few clusters are in that shape. Similarly, despite being better than the DB and the CH in case of the clusters are not well separated, the DI encounters some issues with computational cost when the number of clusters or dimensionality is high. Besides, it is affected by the noisy data due to increasing diameter. As for the S_Dbw, although it is proposed as a density-supported validity index and gets a good score with the compact and well-separated clusters, it is affected by the distribution of the data. In addition, thanks to being a density-based clustering validity index, the DSI is good at dealing with arbitrary-shaped clusters. It can successfully evaluate any cluster quality. However, the DSI is also another cluster validity index that is affected when clusters are too close. Likewise, the RMSSTD is another validity index that encounters some problems when the clusters are close to each other. The examples of the problems on the shapes of clusters that existing indices come across can be increased.

**Figure 4:**
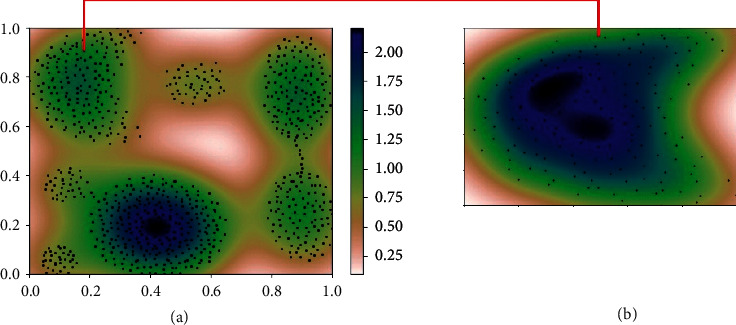
An example of various densities in clusters: example of an Aggregation dataset. (a)Density distribution of the dataset. (b) Density distribution inside a cluster.

Another problem with existing cluster validation indices is that they assume that all the data in any cluster have a homogeneous distribution. However, data inside the cluster mostly have various regions that have different densities, as seen in Figure 4 (darker areas mean denser regions). Moreover, the data in the same cluster may not have homogeneous distribution as can be seen in Figure 4(b). So, any approach that considers the density of data in the clusters is still needed to support the compactness of the cluster. Although the S_Dbw and the DSI are two examples of cluster validity indices that take into consideration the density of clusters, they do not examine the density areas inside the clusters. These kinds of indices are useful to discover the shapes of clusters. However, maybe, some regions are denser than the other regions inside the cluster, and these indices do not take into account such problems. Giving more weight to denser regions may make the approach more accurate while identifying it because of supporting *compactness*. In the present study, we proposed a new cluster validity index that can discover the arbitrary-shaped clusters and weight the denser regions by using the Kernel. Density estimation was explained in Section 4.2.

## 4. Proposed Cluster Validity Index: A Novel Internal Cluster Validity Index for Arbitrary-Shaped Clusters Based on the Kernel Density Estimation (The VIASCKDE Index)

### 4.1. Basic Idea

**Figure 5:**
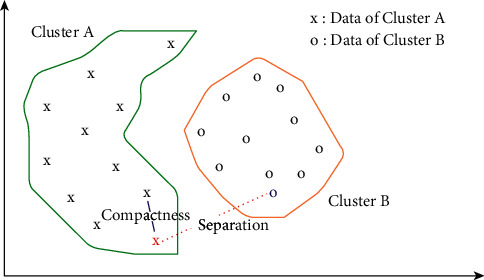
Relationship between the compactness and separation values of any data in the VIASCKDE Index.

In the present study, a new cluster validation index, which has been named shortly the VIASCKDE (the Validity Index for Arbitrary-Shaped Clusters based on the Kernel Density Estimation) index, was proposed. The VIASCKDE Index is a kind of index that is not affected by cluster shape, and thus, it can make a realistic evaluation of clustering performance regardless of the clusters' shape. Unlike the existing cluster validation indices, our index calculates the compactness and separation values of the cluster based on calculating the compactness and separation values for each data separately. In other words, it calculates the compactness and separation values of the cluster over the distance of data, independent of parameters such as the cluster center because, in nonspherical clusters, the distance of the data to the closest data is more important than its distance to the cluster center. As can be seen in the example given in Figure 5, the closest data in the cluster that “*it belongs to”* are used when calculating the compactness value for the data *x*. Similarly, the separation value of *x* is calculated by the distance to the closest data of the cluster that “it does not belong.”

As mentioned before, another problem with existing cluster validity indices is to assume that the distribution of the data inside the cluster has homogeneous distribution, even if the shape of the cluster is arbitrary. Therefore, they weight each data of the cluster as the same value, whereas, as presented in Figure 4, the distribution of data that is inside the same cluster may vary. Therefore, we need a new method that considers this situation. To overcome this problem, we proposed the kernel density estimation (KDE), which is detailed in the next section based on weighting method.

### 4.2. Kernel Density Estimation-Based Weighting

In the literature, there are two types of distribution estimation methods that are parametric and nonparametric. In parametric methods, for example, the Gaussian distribution assumes the distribution of any dataset is gathered around the center and the majority of the data is in a circle having a radius of the standard deviation. It means that the curve has only one peak on distribution. It is important to keep in mind that the univariate normal distribution, with mean *µ* and variance *σ*^*2*^, has the probability density function

(13) $$\begin{array}{c} f\left(x\right)=\frac{1}{\sqrt{2\pi \sigma^{2}}}e^{-{\left[x-\mu /\sigma \right]}^{2}/2}, \end{array}$$

where *x* is in *-∞ < x < ∞* interval. On the other hand, in nonparametric distribution estimation methods, it is assumed that there may be more than one distribution peaks on the curve. Let *X*=[*X*_1_,…,*X*_*n*_]^*T*^ be an *n-*dimensional vector that has a multivariate Gaussian (or normal) distribution with the *n*-dimensional mean vector *μϵR*^*n*^ and ∑ be the *n x n* covariance matrix. The multivariate Gaussian distribution is calculated as follows:

(14) $$\begin{array}{c} p\left(x,\mu ,\Sigma \right)=\frac{1}{\sqrt{{\left(2\pi \right)}^{n}\left|\Sigma \right|}}\text{exp}\left(-\frac{1}{2}{\left(\pi -\mu \right)}^{T}\Sigma^{-1}\left(x-\mu \right)\right). \end{array}$$

**Figure 6:**
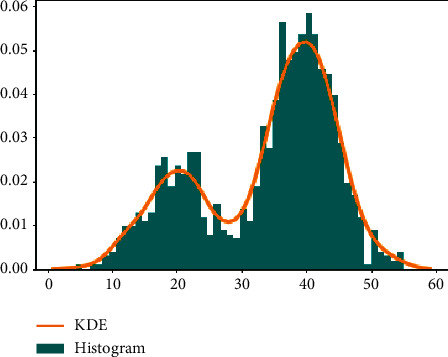
An example of the kernel density estimation and its histogram.

**Figure 7:**
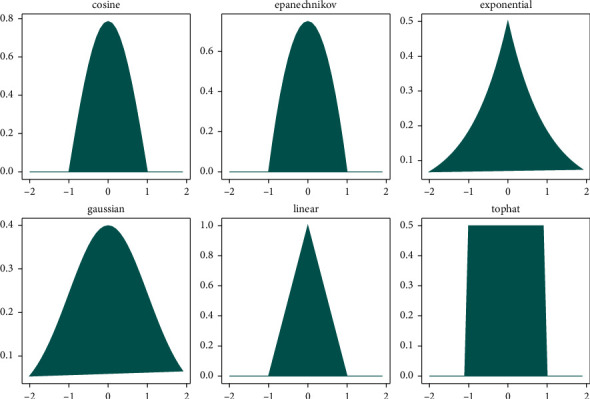
Types of kernel density estimation curves.

The kernel density estimation (KDE) is a nonparametric density estimator that is used for density estimation. It is also a method that is used to analyze existing data to decide which incoming data is placed correctly in which place. For this ability, it is commonly used in many areas such as data analysis procedures in healthcare services, artificial intelligence applications, the stock market, and many other areas [2]. The bar graph represents the histograms, and the orange line represents the KDE, and it is calculated over the histograms as presented in Figure 6. In analyzing the data and representing its application, it figures out the distribution of data according to various methods, which are given in Figure 7. Each one has its characteristic and equation. In mathematical formulation, the KDE is a function

(15) $$\begin{array}{c} \hat{P_{n}}\left(n\right)=\frac{1}{nh}\overset{n}{\underset{i=1}{\sum }}K\left(\frac{X_{i}-x}{h}\right), \end{array}$$

where *K(*.) is one of the functions, which are given in Figure 7. The most commonly used one is the Gaussian function. These functions are known as smooth functions that control the amount of smoothing where the *h* *>* *0*. The KDE smooths each data; here, it is *X*_*i*_, one after the other one until reaching the final density estimation.

In addition to estimating the density function of univariate data, as an example given in Figure 6, we can apply the KDE to multivariate datasets. In this case, we have to use a kernel function that could process a multidimensional dataset. To achieve this, the mentioned kernel function should be constructed by a product kernel or a radial basis approach. Let *X*=(*X*_1_, *X*_2_, *X*_3_,…, *X*_*d*_)′ denote a sample of size *n* from a multivariate random variable with density *f*(*x*) defined on *R*^*d*^, and let {*x*_1_,…, *x*_*n*_} be an independent random sample drawn from *f*(*x*). In the following example, we only considered the two-dimensional case without the loss of generality. Thus, *X*_*i*_,  *i*=1,…, *n*  is given by (*X*_*i*1_, *X*_*i*2_), where *X*_*i*1_ and *X*_*i*2_ denote the *x* and *y* coordinates, respectively. The multivariate kernel density estimator at point *x* is given by

(16) $$\begin{array}{c} \hat{fh}\left(x\right)=\frac{1}{n{\left|h\right|}^{-1/2}}\overset{n}{\underset{i=1}{\sum }}K\left(h^{-1/2}\left(x-X_{i}\right)\right), \end{array}$$

where *K*(.) is a multivariate kernel function and *h* denotes a symmetric positive definite bandwidth matrix.

Although KDE is a nonparametric probability density function to solve the inhomogeneous distribution problem, we can also use it as a weighting function to support the compactness of clusters. As the KDE of any data is the summation of the data around it, it is expected the weight of any data close to the edges of data distribution would be less, while the KDE of the data in the near center would be more. Therefore, the KDE could be used as a weighting function to weight the data. In our approach, doing that will support the compactness of the cluster regardless of its shape. Namely, we used the KDE to weight each data to give more importance to the data in the denser regions. Therefore, we calculated the weight of each data that is *W*_*KDE*_ according to obtained KDE value. For example, let us assume we want to find *W*_*KDE*_ values for data *x*_*1*_ *=* *30 and x*_*2*_ *=* *40* in the example of the dataset given in Figure 6. *W*_*KDE*_ for *x*_*1*_ would be 0.007, while *W*_*KDE*_ would be 0.05 for *x*_*2*_, which is very high when compared to the other one. That makes our approach superior when compared with existing clustering validity indices, which ignore the distribution of data in the same cluster. In other density-based approaches, they would weight *x*_*1*_ and *x*_*2*_ as equal for this example and this would be incorrect.

### 4.3. Definitions and Equations

In light of these explanations, let us explain the details of the VIASCKDE Index.

**Definition 1 .**

(*CoSeD—Compactness and Separation Value of a Data*): the *CoSeD* can be described as the compactness and separation value of any data. To calculate this value, *W*_*KDE*_ value of each data, which is explained in Section 4.2, is calculated first. Let *a( x *) (*compactness*) be the distance from *x* to the closest data of cluster *C*_*i*_ in which the data *x* also belong, and let *b( x *) (*separation*) be the distance from *x* to the closest data of cluster *C*_*j*_ in which the data *x* do not belong to; therefore, the compactness and separation value of the data *x*, *CoSeD( x *), are calculated by the following equation:

(17) $$\begin{array}{c} a\left(x\right)={\text{min}}_{x\in C_{i},\;y\in C_{i}}\left\{d_{e}\left(x,y\right)\right\},\\ b\left(x\right)={\text{min}}_{x\in C_{i},\;y\in C_{j},\;x\neq y}\left\{d_{e}\left(x,y\right)\right\},\\ CoSeD\left(x\right)=W_{KDE}\frac{b\left(x\right)-a\left(x\right)}{\text{max}\left\{a\left(x\right),\;b\left(x\right)\right\}}. \end{array}$$

**Definition 2 .**

(*CoSeC—Compactness and Separation Value of a Cluster*): the *CoSeC* value is the average of the CoSeD values of the data owned by the cluster. The CoSeC value of the cluster *C*_*i*_ is calculated by equation (18), where *C*_*i*_ is the cluster to which the data *x* belong, and *n* is the number of the data that cluster *C*_*i*_ possesses.

(18) $$\begin{array}{c} CoSeC\left(C_{i}\right)=\;\frac{1}{n}\overset{n}{\underset{i=1}{\sum }}CoSeD\left(x_{i}\right). \end{array}$$

**Definition 3 .**

(the *VIASCKDE*, *the Value of Overall Clustering*): let *k* be the number of clusters, let *n*_*j*_ be the number of data that cluster *C*_*j*_ possesses, and let *CoSeC*_*j*_ be the value of cluster *C*_*j*_, which is calculated in equation (18); therefore, the VIASCKDE Index value is calculated by equation (19). The VIASCKDE value is expected to be in between [−1, +1], where +1 refers to the best possible value, and -1 refers to the worst possible value.

(19) $$\begin{array}{c} VIASCKDE=\frac{\sum_{j=1}^{k}n_{j}CoSeC_{j}}{\sum_{j=1}^{k}n_{j}}. \end{array}$$

### 4.4. The Algorithm

Let Gaussian_KDE be a function that calculates the KDE and MinMaxNormalization, which is also a function that normalizes the data to the range of [0, 1]. The CoSeD and CoSeC values were explained in Section 4.3. In light of this information and the equation given in the previous section, the pseudocode of VIASCKDE Index was given in Algorithm 1.

### 4.5. Computational Complexity

Let *k* be the number of clusters in the dataset, let *n* be the number of data that clusters possess, and let *d* be the number of features each data possesses; therefore, the time complexity of the VIASCKDE Index is calculated as the O(*kn*^*2*^*d*), since it calculates the distance of each data to all others. This means that the complexity of the proposed approach is the O(*n*^*2*^). This is acceptable when the index is compared with the complexity of other indices given in Table 1.

## 5. Experimental Study

### 5.1. Development Environment

To demonstrate the effectiveness of the VIASCKDE Index (https://github.com/senolali/VIASCKDE) on the experimental studies, the data were processed with using the *Python* language in the Anaconda Spyder environment. Various machine learning libraries of the Scikit-learn library such as the DBSCAN, Spectral Clustering, HDBSCAN, and metrics were used. The dataset was imported with the Pandas library, and mathematical operations were performed with the NumPy library. Visualization processes were also carried out with the matplotlib library. All experiments and comparison operations were performed on a computer with 16 GB RAM, Intel i7 processor, and Windows 11 operating system.

### 5.2. Used Datasets

**Figure 8:**
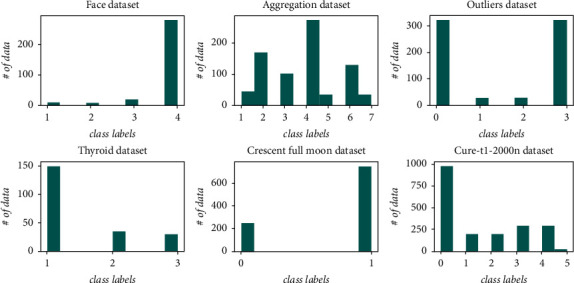
The distributions of some of the used datasets.

To measure the performance of the proposed approach, we performed an experimental study in both synthetic and real datasets. Since the main purpose of our approach is to measure the performance of nonspherical clusters, artificial datasets containing clusters in different shapes were used. In Figure 3, some of the used datasets that contain clusters in different shapes are demonstrated. In addition to these synthetic datasets, real datasets, which are frequently used in the clustering field, were also used for testing. Details of the datasets used in the comparison process are provided in Table 2. Additionally, as given in Figure 8, some imbalanced datasets were used to analyze the performance of our cluster validation index on the imbalanced data distribution.

### 5.3. Experimental Procedure

For the experimental study, we used the procedure given below. But firstly, to ensure that each data are between the same ranges and to make it easy to determine parameters, the data were normalized using the min-max normalization that was demonstrated in (20). In addition, the ARI (Adjusted Rand Index) was used as the ground truth method to evaluate the performance of cluster validation indices by comparing the cluster labels that were produced by the clustering algorithm with the actual cluster labels. The reason we chose the ARI is that the generated cluster labels do not need to be the same as the actual cluster labels. For example, let us assume the clustering algorithm produced {1,1,1,2,2,2} cluster labels and actual labels are {2,2,2,4,4,4}. The accuracy value for this situation would be 0%, while it would be 100% with the ARI value, which should be the actual result.

(20) $$\begin{array}{c} z_{ij}=\frac{x_{ij}-\text{min}x_{j}}{\max x_{j}-\min x_{j}}. \end{array}$$

The procedure established in the testing process is as follows:

  Step #1:
  Select one of the algorithms (DBSCAN,HDBSCAN, and Spectral Clustering)
  Step #2:
  Test the algorithm with randomly selected parameters on one of the selected datasets.
  Step #3:
  Evaluate the cluster qualities of clusters that were produced by the selected algorithm with clustering validation indices (SI, DI, CH, DB, S_Dbw, DSI, RMSSTD, and VIASCKDE).
  Step #4:
  Calculate the VIASCKDE Index via produced clusters and evaluate it to see whether this is the best result so far. If it is, we accept this value as the best one for the VIASCKDE Index. Then, we do the same operation for the other indices.
  Step #5:
  To test each index sufficiently, go to Step #2 and repeat the cycle 100 times. If the cycle is completed go to Step #6.
  Step #6:
  Calculate the ARI value that corresponds to the most successful value obtained for each of the clustering validity indices including our proposed approach.
  Step #7:
  Compare the ARI values calculated by all cluster validity indices. Consider the one with the highest ARI value as the most competent one for this dataset.
  Step #8:
  Go to Step 2 and do the same operations for the new dataset. If all datasets are performed, go to Step 9.
  Step #9:
  If all algorithms are performed, finish the procedure; otherwise, go to Step 1.

### 5.4. Experimental Study

#### 5.4.1. The Selection of Density Distribution Estimation Method

We performed some experimental studies on the datasets to decide which data distribution method should be selected, either parametric or nonparametric. For the parametric method, we selected the Gaussian method and the KDE for the nonparametric method. We carried out experiments with the procedure given in Section 5.3, by using the DBSCAN in which the parameters are randomly selected. Besides, the *kernel* *=* “*Gaussian*” and *h* *=* *0*.*05* were the parameters of KDE based on the VIASCKDE Index approach, while the Gaussian was the method of parametric VIASCKDE Index. According to obtained results, while the Gaussian-based method outperformed in 15 datasets, the KDE-based method was the best in 17 datasets, as demonstrated in Table 3. Therefore, we selected the KDE-based method as the weighting function for our approach.

#### 5.4.2. The Kernel Selection for KDE

As mentioned in Section 4.2, there are various kernels in the literature. The Gaussian, cosine, linear, tophat, and exponential can be given as examples, and they affect the smoothness of distribution. We fulfilled the operation with the procedure provided in Section 5.3 where the parameters of DBSCAN algorithm were selected randomly. We performed the experiments by choosing each kernel in each experimental study. As it can be seen in Table 4, the Gaussian kernel was the best in all of the selected datasets, when the bandwidth was *0*.*05*.

#### 5.4.3. Bandwidth Selection for the KDE

**Figure 9:**
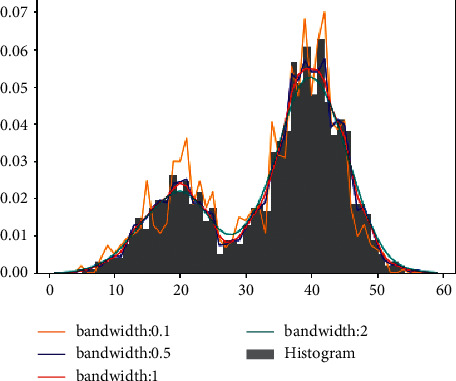
Types of the kernel density estimation curves.

One of the most important parameters of KDE is bandwidth (*h*). It possesses a direct effect on the results. When the *h* is too small, there would be many wiggly structures on the density curve. On the other hand, when the *h* is too large, the bumps on the curve would be smoothed out as given in Figure 9. To find which bandwidth is the best for our approach, we fulfilled some experimental studies with the procedure given in Section 5.3 by testing it with different bandwidth values on some datasets, which are provided in Table 2. The best bandwidth was found to be 0.05 as it can be seen in Table 5, when the kernel was the Gaussian.

#### 5.4.4. The Tests on Both Synthetic and Real Datasets

**Figure 10:**
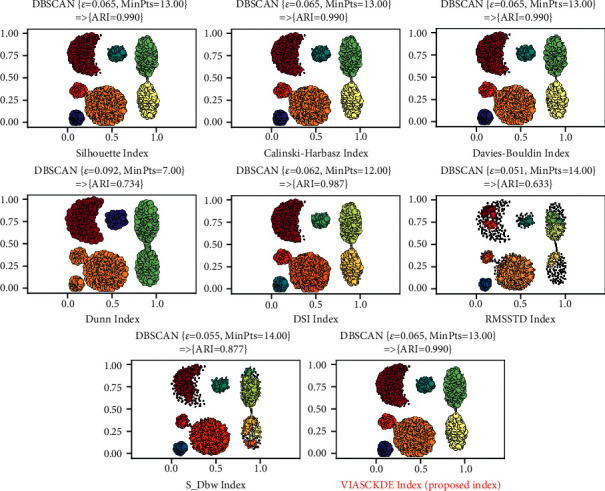
The clustering results suggested by each validity index when the DBSCAN algorithm was tested in the Aggregation dataset.

In this section, experimental works were executed on both synthetic and real datasets. To detect nonspherical clusters in the test process, the DBSCAN, Spectral Clustering, and HDBSCAN were used. The DBSCAN algorithm uses two parameters (*MinPts*: the clustering threshold value, and *ε*: the accessibility distance) and Spectral Clustering uses one parameter as input (*n_clusters*: the number of clusters) if the *affinity* *=*  *“nearest_neighbors*,” while the HDBSCAN Clustering uses two parameters (*min_cluster_size*: the number of clusters, and *min_samples*). To test each algorithm with different parameters, we performed the random search method on the procedure given in Section 5.3. The procedure given above with each cluster validity index was used as the leading method to reach better clustering results. As an example is given in Figure 10, each index proposed various results. It means that the cluster validation performance of each one is also different. According to obtained results, our index was the best one. The performance of each index in all datasets is presented in the following tables for each clustering algorithm (Tables 6–14).

## 6. Evaluation of the Results and Discussion

In our approach, we used the compactness and separation values of each data to support the arbitrary-shaped clusters. In this case, our approach tended to divide the spherical clusters into small partitions. To cope with this issue, we used a density estimation method to support the compactness of clusters. In the literature, there are two types of density estimation methods, parametric and nonparametric methods. To decide which one is the best for our approach, we carried out some experiments on the datasets by using the DBSCAN as the clustering algorithm. According to the experimental study, the nonparametric method was better than the parametric method, and the results of it can be seen in Table 3. After deciding that the nonparametric method was the best for our approach, we selected the kernel density estimation as the nonparametric density estimation method in order to support the multivariate (Table 4).

The second point worth discussing is the selection of parameters of the kernel density estimation. The kernel density estimation has two parameters: the first one is the kernel method and the second one is the bandwidth. To find the best parameters of the kernel density estimation, we conducted some experimental studies. We carried out separate experiments for each parameter by using the procedure given in Section 5.3 by using the DBSCAN with randomly selected parameters. As it can be seen in Tables 4 and 5, *the Gaussian* was the best kernel method and the *h* *=* *0*.*05* was the best bandwidth. These parameters were the parameters that were used in experimental studies, which were used to compare our approach with the other indices.

One of the advantages of the proposed VIASCKDE Index is that it can realistically evaluate the clustering performance regardless of the cluster shape. To test the success of our index on different cluster types, we used the DBSCAN, Spectral Clustering, and HDBSCAN algorithms with the procedure given in Section 5.3. The highest ARI values found as the best value by each index are given in Tables 11, 12 and 14. As it can be seen in the tables, the VIASCKDE Index reaches the highest ARI values on most of the datasets. The VIASCKDE Index reaches the highest ARI values in 47 of the 60 experiments, as given in Table 15. In addition, the ARI value of our index was very high, even if it was not the index that had the highest ARI value. In addition, when our index was compared with the density-based two indices, which were the S_Dbw and DSI, better results were obtained, and they are demonstrated in Table 15.

The other important advantage of our approach is that it considers the density of each cluster independently. For example, the Aggregation dataset has a nonhomogeneous density as it can be seen in Figure 4, and each cluster also may have a nonhomogeneous distribution as it was given in Figure 4(b). So, our approach does not assume all data inside any cluster has homogeneous distribution and also does not weight each data equally. It gives more importance to the data in the denser regions by multiplying those data with a coefficient that is detected by the KDE. Doing that supports the compactness of clusters. In other words, this approach made our index got better results.

Since the VIASCKDE Index has a density-based approach, it can also be used to evaluate the performance of the algorithms that are based on a microcluster structure, which is used by the majority of density-based clustering algorithms because such algorithms use the center of each of the microclusters as the actual data in the offline phase. Therefore, the VIASCKDE Index can also be used to evaluate the performance of micro-cluster-based clustering algorithms.

## 7. Conclusion and Future Works

In the present study, we proposed a cluster validation index, which is called the VIASCKDE Index to validate the clusters quality of both the spherical and nonspherical clusters. Our approach draws its strength from considering the distribution of data inside the clusters by using the KDE. Doing that supports the compactness of clusters irrespective of the cluster center, and thus, the shape of the cluster can be in the form of arbitrary cluster. Most of the cluster validity indices in the literature can only do a realistic cluster quality evaluation when the cluster shape is spherical. However, in many instances, the cluster shape is not spherical. Our proposed approach calculates the compactness and separation values only based on the data. This approach makes it possible to evaluate cluster quality irrespective of its shape. Experimental studies revealed that the VIASCKDE Index reached the highest ARI values in most of the datasets. This means that the approach we proposed is the most successful one among the others. It has been planned to carry out studies to decrease the runtime complexity of the proposed index in the future.

## Figures and Tables

**Algorithm 1:**
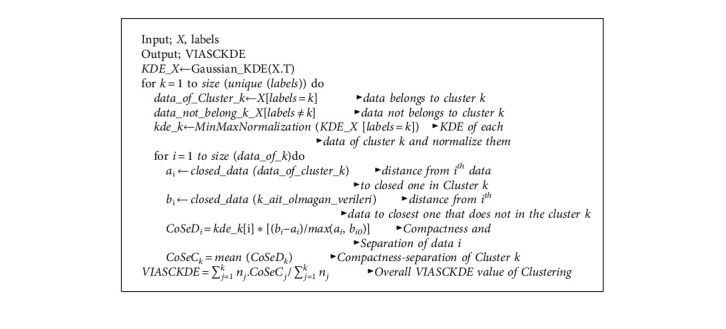
VIASCKDE Index.

**Table 1:** Comparison of clustering validity indices that were used for experimentation in the present study.

Cluster validity Index	Notation	Runtime complexity	Optimal value	Considering denser region?	Handling arbitrary-shaped clusters?	Advantages	Disadvantages
Silhouette Index [30]	SI	*O*(*n*^2^)	Max.	✗	✗	The score is higher when the clusters are dense and well separated	Good at handling the spherical clusters, high computational complexity
Dunn Index [31]	DI	*O*(*n*^2^)	Max.	✗	✓	Competent at cluster validity task	High computational cost with high-dimensional data and the number of clusters
Calinski-Harabasz Index [33]	CH	*O*(*n*)	Max.	✗	✗	Good at well separated and compact clusters, its computational complexity is very low	It is not competent enough at the cluster validation task.
Davies–Bouldin Index [32]	DB	*O*(*n*)	Min.	✗	✗	Good at well separated and compact clusters, its computational complexity is very low	It is not competent enough at the cluster validation task.
S_Dbw validity Index [35]	S_Dbw	*O*(*n*)	Min.	✗	✓	Its computational complexity is very low	Affected negatively by the distribution of data
Distance-based Separability Index [39]	DSI	*O*(*n*^3^)	Min	✗	✓	Useful to discover the shape of clusters	Affected negatively when clusters are too close and its computational complexity is high
Root-mean-square std dev [35]	RMSSTD	*O*(*n*)	Min.	✗	✗	Good for hierarchical clustering	Has issues when the clusters are close to each other
VIASCKDE Index (proposed)	VIASCKDE	*O*(*n*^2^)	Max.	✓	✓	It can handle the arbitrary-shaped clusters, take into account the denser regions, can be used for density-based and micro-cluster-based approaches	Has issues when the clusters are close to each other

**Table 2:** Used datasets.

Dataset	Type	# of Features	# of data	# of classes	Reference
Half-kernel	Synthetic	2	1000	2	[45]
Two spirals	Synthetic	2	312	3	[45]
Outlier	Synthetic	2	700	4	[45]
Corners	Synthetic	2	2000	4	[45]
Cluster in cluster	Synthetic	2	1012	2	[45]
Crescent full moon	Synthetic	2	1000	2	[45]
Moon	Synthetic	2	514	4	[45]
Face	Synthetic	2	322	4	[46]
Wave	Synthetic	2	287	2	[46]
Aggregation	Synthetic	2	788	7	[47]
Zelnik1	Synthetic	2	622	4	[48]
Zelnik5	Synthetic	2	512	4	[48]
Xclara	Synthetic	2	3000	3	[48]
Banana	Synthetic	2	4811	2	[48]
D2c2sc13	Synthetic	2	588	13	[48]
2sp2glob	Synthetic	2	999	3	[48]
Cure-t1-200n	Synthetic	2	2000	5	[48]
Thyroid	Real	4	215	2	[49]
Fisher iris	Real	4	150	3	[49]
Breast cancer	Real	8	699	2	[49]

**Table 3:** ARI results obtained with the parametric and nonparametric methods.

Datasets	Adjusted Rand Index (ARI)	
	Methods	
	Gaussian Weight	KDE Weight
Half-kernel	**1.0000**	**1.0000**
Two spirals	**1.0000**	**1.0000**
Outlier	**1.0000**	**1.0000**
Corners	**1.0000**	**1.0000**
Cluster in cluster	**1.0000**	**1.0000**
Crescent full moon	**1.0000**	**1.0000**
Moon	**0.7424**	**0.7424**
Face	0.9949	**1.0000**
Wave	1.0000	**1.0000**
Fisher iris	0.7493	**0.7493**
Breast cancer	0.7540	**0.7540**
Aggregation	0.7338	**0.9118**
Thyroid	-0.0619	**0.6783**
Zelnik1	**1.0000**	0.9488
Zelnik5	**1.0000**	**1.0000**
Xclara	0.0001	0.0001
Banana	**1.0000**	**1.0000**
Ds2c2sc13	0.3187	**0.5904**
2sp2glob	**1.0000**	0.9880
Cure-t1-2000n	**0.8850**	**0.8850**

**Table 4:** Obtained results with the different kernels values.

Kernels	Datasets											
	Obtained VIASCKDE Values with each *kernel*						Obtained ARI Values with each *kernel*					
	Face	Aggregation	Outliers	Thyroid	Crescent full moon	Cure-t1-200n	Face	Aggregation	Outliers	Thyroid	Crescent full moon	Cure-t1-200n
Gaussian	0.7063	0.6368	0.6797	0.4947	0.6623	0.6555	**0.6085**	**0.8246**	**1.0000**	**0.5083**	**1.0000**	**0.8850**
Cosine	0.5967	0.6564	0.6499	0.1699	0.6340	0.6343	**0.6085**	0.8089	**1.0000**	**0.5083**	**1.0000**	**0.8850**
Exponential	0.7005	0.6371	0.6714	0.5541	0.6426	0.6653	0.0386	0.8089	**1.0000**	0.5034	**1.0000**	**0.8850**
Linear	0.5736	0.6427	0.6306	0.1594	0.6169	0.6371	**0.6085**	0.8089	**1.0000**	**0.5083**	**1.0000**	**0.8850**
Epanechnikov	0.6021	0.6562	0.6581	0.1758	0.6388	0.6295	**0.6085**	0.8089	**1.0000**	**0.5083**	**1.0000**	**0.8850**
Tophat	0.6457	0.6165	0.6433	0.2306	0.6664	0.6299	**0.6085**	0.0333	**1.0000**	**0.5083**	**1.0000**	**0.8850**

**Table 5:** Obtained results with the different bandwidth values.

Bandwidth	Datasets											
	Obtained VIASCKDE values with each *bandwidth*						Obtained ARI values with each *bandwidth*					
	Face	Aggregation	Outliers	Thyroid	Crescent full moon	Cure-t1-200n	Face	Aggregation	Outliers	Thyroid	Crescent full moon	Cure-t1-200n
0.01	0.3377	0.3444	0.4650	0.0556	0.4780	0.5264	−0.0386	0.8089	**1.0000**	**0.5277**	**1.0000**	**0.8850**
0.03	0.6627	0.6565	0.6508	0.3493	0.6608	0.6421	**0.6085**	0.8089	**1.0000**	0.5034	**1.0000**	**0.8850**
0.05	0.7063	0.6388	0.6797	0.4947	0.6623	0.6555	**0.6085**	**0.9898**	**1.0000**	0.5034	**1.0000**	**0.8850**
0.1	0.7365	0.6225	0.6851	0.6306	0.6486	0.6565	−0.0386	0.8089	**1.0000**	0.5034	**1.0000**	**0.8850**
0.3	0.7857	0.5947	0.6773	0.7402	0.6143	0.6189	−0.0386	0.7338	**1.0000**	0.2099	**1.0000**	**0.8850**
0.5	0.7586	0.5689	0.5481	0.7591	0.5945	0.6039	−0.0386	0.7338	**1.0000**	0.2099	**1.0000**	**0.8850**
1.0	0.7412	0.5636	0.5257	0.7618	0.5927	0.6018	−0.0386	0.7338	**1.0000**	0.2099	**1.0000**	**0.8850**
1.5	0.7362	0.5629	0.5236	0.7618	0.5923	0.6016	−0.0386	0.7338	**1.0000**	0.2099	**1.0000**	**0.8850**
2	0.7339	0.5626	0.5229	0.7618	0.5921	0.6015	−0.0386	0.7338	**1.0000**	0.2099	**1.0000**	**0.8850**
2.5	0.7328	0.5625	0.5226	0.7618	0.5920	0.6015	−0.0386	0.7338	**1.0000**	0.2099	**1.0000**	**0.8850**
3	0.7322	0.5624	0.5225	0.7618	0.5920	0.6015	−0.0386	0.7338	**1.0000**	0.2099	**1.0000**	**0.8850**
3.5	0.7317	0.5624	0.5223	0.7618	0.5919	0.6015	−0.0386	0.7338	**1.0000**	0.2099	**1.0000**	**0.8850**
4	0.7314	0.5623	0.5222	0.7617	0.5919	0.6015	−0.0386	0.7338	**1.0000**	0.2099	**1.0000**	**0.8850**
4.5	0.3377	0.3444	0.4650	0.0556	0.4780	0.5264	−0.0386	0.8089	**1.0000**	**0.5277**	**1.0000**	**0.8850**
5	0.6627	0.6565	0.6508	0.3493	0.6608	0.6421	**0.6085**	0.8089	**1.0000**	0.5034	**1.0000**	**0.8850**

**Table 6:** The best parameters for datasets that were detected by the cluster validity indices with the DBSCAN algorithm.

Dataset	DBSCAN parameters	Best parameters detected by indices for the DBSCAN algorithm							
		SI	DI	DB	CH	S_Dbw	DSI	RMSSTD	VIASCKDE
Half-kernel	*ε*	0.08	0.08	0.05	0.08	0.05	0.05	0.08	0.08
	MinPts	7	7	11	7	15	11	7	7
Two spirals	*ε*	0.1	0.1	0.05	0.1	0.05	0.1	0.05	0.1
	MinPts	11	11	15	11	15	11	14	11
Outlier	*ε*	0.07	0.07	0.07	0.07	0.05	0.07	0.05	0.07
	MinPts	15	15	15	15	8	15	14	15
Corners	*ε*	0.1	0.1	0.1	0.1	0.1	0.1	0.1	0.1
	MinPts	15	15	15	15	15	15	15	15
Cluster in cluster	*ε*	0.06	0.06	0.06	0.06	0.06	0.06	0.06	0.06
	MinPts	12	12	12	12	12	12	14	12
Crescent full moon	*ε*	0.07	0.07	0.07	0.07	0.05	0.06	0.05	0.07
	MinPts	14	14	14	14	15	12	15	14
Moon	*ε*	0.06	0.08	0.06	0.06	0.05	0.05	0.06	0.06
	MinPts	7	11	9	7	9	9	15	15
Face	*ε*	0.06	0.1	0.1	0.06	0.06	0.05	0.06	0.1
	MinPts	15	8	5	6	15	12	11	8
Wave	*ε*	0.09	0.09	0.06	0.09	0.05	0.06	0.05	0.06
	MinPts	12	5	12	12	9	12	15	12
Fisher iris	*ε*	0.14	0.19	0.14	0.14	0.08	0.14	0.06	0.19
	MinPts	15	6	15	15	5	15	7	6
Breast cancer	*ε*	0.39	0.33	0.39	0.39	0.06	0.06	0.05	0.4
	MinPts	8	5	8	8	5	5	14	5
Aggregation	*ε*	0.06	0.09	0.06	0.06	0.06	0.06	0.05	0.06
	MinPts	13	7	13	13	14	12	14	13
Thyroid	*ε*	0.1	0.1	0.06	0.09	0.07	0.05	0.05	0.1
	MinPts	5	5	12	5	6	8	9	5
Zelnik1	*ε*	0.08	0.08	0.05	0.1	0.07	0.07	0.08	0.07
	MinPts	6	15	14	7	5	5	15	5
Zelnik5	*ε*	0.06	0.1	0.05	0.1	0.06	0.05	0.05	0.1
	MinPts	14	13	12	13	15	12	14	13
Xclara	*ε*	0.05	0.08	0.09	0.05	0.05	0.05	0.08	0.05
	MinPts	13	12	15	13	13	13	12	13
Banana	*ε*	0.05	0.05	0.05	0.05	0.05	0.05	0.05	0.05
	MinPts	9	9	9	9	9	9	9	9
Ds2c2sc13	*ε*	0.09	0.09	0.06	0.06	0.05	0.06	0.09	0.05
	MinPts	10	10	14	14	13	14	10	8
2sp2glob	*ε*	0.1	0.1	0.05	0.07	0.08	0.1	0.06	0.07
	MinPts	9	9	12	14	6	9	5	14
Cure-t1-2000n	*ε*	0.1	0.1	0.1	0.1	0.1	0.1	0.1	0.1
	MinPts	10	10	10	10	10	10	10	10

**Table 7:** Obtained values for each index based on the parameters given in Table 6.

Dataset	Obtained values for the each index							
	SI	DI	DB	CH	S_Dbw	DSI	RMSSTD	VIASCKDE
Half-kernel	0.2010	0.0949	1.8818	127.8905	0.5419	0.5068	0.2495	0.7125
Two spirals	0.0588	0.1317	3.3241	152.9447	0.5848	0.1069	0.28	0.7903
Outlier	0.5608	0.4291	0.4037	1075.5609	0.2099	0.9654	0.1302	0.6797
Corners	0.4614	0.2872	0.7436	2020.1068	0.4976	0.6358	0.1187	0.6295
Cluster in cluster	0.2231	0.2341	208.8458	0.0169	0.8536	0.7332	0.2276	0.595
Crescent full moon	0.2784	0.1923	1.1646	285.1423	0.3255	0.6568	0.2449	0.6623
Moon	0.2371	0.1052	0.9739	244.1722	0.2081	0.8788	0.2525	0.7508
Face	0.4569	0.2217	1.1099	213.0246	0.3725	0.7627	0.2423	0.6631
Wave	0.4525	0.1291	0.7119	366.1095	0.2344	0.8935	0.2696	0.6495
Fisher iris	0.5692	0.1222	0.5234	223.6137	0.3386	0.8296	0.2527	0.443
Breast cancer	0.5698	0.1228	0.8037	900.1988	0.3606	0.9617	0.2993	0.2944
Aggregation	0.4763	0.1432	0.5461	1156.7539	0.2073	0.9442	0.1878	0.6388
Thyroid	0.433	0.0598	2.7626	16.6429	0.5343	0.7486	0.1528	0.3275
Zelnik1	0.2045	0.0992	5.6978	95.196	0.2523	0.8939	0.2171	0.6604
Zelnik5	0.4971	0.2224	0.8098	413.8835	0.3651	0.8338	0.1534	0.7739
Xclara	0.6654	0.0656	1.1863	6889.0154	0.3492	0.7462	0.229	0.8101
Banana	0.3589	0.1258	1.1322	3532.2201	0.7625	0.4334	0.2146	0.8076
Ds2c2sc13	0.5724	0.237	0.5891	1907.2388	0.1921	0.9193	0.1091	0.605
2sp2glob	0.3899	0.1278	2.7559	158.5187	0.6374	0.8003	0.2089	0.8819
Cure-t1-2000n	0.4514	0.1196	0.6775	1365.0774	0.3054	0.787	0.1721	0.6555

**Table 8:** The best parameters for the datasets that were detected by the cluster validity indices with the Spectral Clustering algorithm are given in Table 7.

Dataset	Spectral clustering parameters	Best parameters detected by indices for the Spectral Clustering algorithm							
		SI	DI	DB	CH	S_Dbw	DSI	RMSSTD	VIASCKDE
Half-kernel	n_clusters	14	2	15	15	14	15	2	2
Two spirals	n_clusters	15	2	15	15	15	15	2	2
Outlier	n_clusters	2	4	4	13	3	4	2	4
Corners	n_clusters	12	4	12	12	15	14	2	2
Cluster in cluster	n_clusters	4	2	4	15	15	15	2	2
Crescent full moon	n_clusters	5	2	5	13	15	14	2	6
Moon	n_clusters	15	2	15	15	15	15	2	2
Face	n_clusters	11	2	10	12	15	13	2	2
Wave	n_clusters	7	2	15	15	15	15	2	2
Fisher iris	n_clusters	2	2	2	3	15	2	2	3
Breast cancer	n_clusters	2	2	2	2	11	14	15	12
Aggregation	n_clusters	4	2	6	14	2	15	2	2
Thyroid	n_clusters	3	2	3	3	15	15	2	3
Zelnik1	n_clusters	12	2	13	12	15	13	3	3
Zelnik5	n_clusters	8	2	8	15	15	15	2	4
Xclara	n_clusters	3	2	3	3	10	3	2	3
Banana	n_clusters	9	2	9	15	14	15	2	2
Ds2c2sc13	n_clusters	3	3	5	8	2	15	2	5
2sp2glob	n_clusters	7	2	15	15	15	15	2	7
Cure-t1-2000n	n_clusters	5	2	4	13	2	12	2	3

**Table 9:** The best parameters for the datasets that were detected by the cluster validity indices with the HDBSCAN algorithm.

Dataset	HDBSCAN Parameter	Best parameters detected by the indices for the HDBSCAN algorithm							
		SI	DI	DB	CH	S_Dbw	DSI	RMSSTD	VIASCKDE
Half-kernel	n_clusters_size	24	24	2	25	25	25	24	24
	n_samples	6	6	10	25	25	25	6	6
Two spirals	n_clusters_size	3	25	3	17	2	2	15	6
	n_samples	2	17	2	7	2	2	19	12
Outlier	n_clusters_size	16	16	16	16	16	16	16	16
	n_samples	12	12	12	12	12	12	12	12
Corners	n_clusters_size	8	8	8	8	2	2	8	8
	n_samples	8	8	8	8	2	2	8	8
Cluster in cluster	n_clusters_size	20	20	9	11	7	7	20	20
	n_samples	10	10	2	2	3	3	10	10
Crescent full moon	n_clusters_size	20	20	3	20	3	3	20	20
	n_samples	12	12	2	12	2	2	12	12
Moon	n_clusters_size	22	6	22	22	10	2	10	6
	n_samples	3	4	3	3	24	25	24	4
Face	n_clusters_size	21	13	9	21	9	9	13	9
	n_samples	5	19	8	5	8	8	19	8
Wave	n_clusters_size	16	6	16	16	3	4	6	2
	n_samples	13	3	23	13	13	19	3	5
Fisher iris	n_clusters_size	5	5	14	5	5	18	9	5
	n_samples	12	12	16	12	12	21	25	12
Breast cancer	n_clusters_size	11	5	2	5	2	2	22	5
	n_samples	34	55	3	55	3	3	53	55
Aggregation	n_clusters_size	17	12	9	12	23	2	12	2
	n_samples	25	14	16	14	13	4	14	4
Thyroid	n_clusters_size	3	2	3	3	3	3	2	8
	n_samples	2	7	2	2	4	2	16	4
Zelnik1	n_clusters_size	11	3	5	3	20	2	14	3
	n_samples	16	11	25	15	16	17	19	11
Zelnik5	n_clusters_size	20	20	20	20	20	20	20	20
	n_samples	3	3	3	3	3	3	3	3
Xclara	n_clusters_size	9	22	3	13	3	3	3	13
	n_samples	2	6	3	9	3	3	3	9
Banana	n_clusters_size	21	21	13	21	21	16	21	21
	n_samples	14	14	16	14	14	24	14	14
Ds2c2sc13	n_clusters_size	22	22	16	22	4	22	24	16
	n_samples	19	19	20	19	6	19	24	10
2sp2glob	n_clusters_size	21	21	21	21	21	21	21	21
	n_samples	22	22	22	22	22	22	22	22
Cure-t1-2000n	n_clusters_size	4	4	4	4	4	4	4	25
	n_samples	6	6	6	6	6	6	6	4

**Table 10:** Obtained values for each index based on the parameters are given in Table 8.

Dataset	Obtained values for the each index							
	SI	DI	DB	CH	S_Dbw	RMSSTD	DSI	VIASCKDE
Half-kernel	0.4748	0.0949	0.6066	1761.6198	0.2246	0.9163	0.2495	0.7395
Two spirals	0.3175	0.1317	1.058	1378.878	0.2857	0.7829	0.2865	0.8151
Outlier	0.6178	0.4291	0.4037	1804.463	0.1176	0.9654	0.2924	0.6863
Corners	0.5672	0.2872	0.5315	4102.5883	0.1873	0.9439	0.207	0.6575
Cluster in cluster	0.4547	0.2341	0.9465	832.9385	0.2764	0.857	0.2275	0.6052
Crescent full moon	0.4993	0.1923	0.5792	2022.7022	0.2055	0.9103	0.2423	0.6689
Moon	0.4543	0.1285	0.6781	602.0907	0.2169	0.9098	0.2689	0.7527
Face	0.4996	0.2361	0.5473	1055.0573	0.1705	0.9271	0.2481	0.7575
Wave	0.4957	0.1291	0.631	681.3681	0.1639	0.9124	0.2541	0.617
Fisher iris	0.6295	0.3581	0.4877	356.289	0.2163	0.8923	0.1432	0.4539
Breast cancer	0.5839	0.1291	0.7738	993.0158	0.1796	0.7795	0.2031	0.4341
Aggregation	0.4541	0.1091	0.589	1623.9684	0.1434	0.921	0.2966	0.6944
Thyroid	0.5517	0.0973	0.85	138.1291	0.3809	0.685	0.1309	0.4832
Zelnik1	0.5042	0.0992	0.663	194.586	0.2836	0.8614	0.2171	0.6544
Zelnik5	0.5948	0.2651	0.5353	1832.5626	0.1548	0.9495	0.2763	0.7686
Xclara	0.6946	0.023	0.4203	10843.7203	0.2779	0.946	0.1612	0.8164
Banana	0.5087	0.1258	0.5734	14012.5597	0.1806	0.9343	0.2146	0.82
Ds2c2sc13	0.3939	0.0639	0.8082	1133.5545	0.1434	0.9064	0.2896	0.6187
2sp2glob	0.6102	0.1456	0.6921	1548.8465	0.2544	0.8693	0.2396	0.725
Cure-t1-2000n	0.4994	0.1921	0.6581	3615.5302	0.1582	0.9016	0.2817	0.6589

**Table 11:** ARI values were obtained from the parameters that are given in Table 6 and were proposed by each index.

Dataset	Obtained ARI values for the each index							
	SI	DI	DB	CH	S_Dbw	DSI	RMSSTD	VIASCKDE
Half-kernel	**1.0000**	**1.0000**	0.9940	**1.0000**	0.9153	0.9940	**1.0000**	**1.0000**
Two spirals	**1.0000**	**1.0000**	0.9804	**1.0000**	0.9804	**1.0000**	0.9990	**1.0000**
Outlier	**1.0000**	**1.0000**	**1.0000**	**1.0000**	0.9973	**1.0000**	0.8621	**1.0000**
Corners	**1.0000**	**1.0000**	**1.0000**	**1.0000**	**1.0000**	**1.0000**	**1.0000**	**1.0000**
Cluster in cluster	**1.0000**	**1.0000**	**1.0000**	**1.0000**	**1.0000**	**1.0000**	0.8879	**1.0000**
Crescent full moon	**1.0000**	**1.0000**	0.9968	**1.0000**	0.9105	0.9873	0.8509	**1.0000**
Moon	**0.9379**	0.6322	0.9256	**0.9379**	0.7874	0.7874	0.7949	0.7949
Face	0.2645	0.9949	**0.9961**	0.2892	0.1304	0.1226	0.8521	**0.9961**
Wave	0.3514	**1.0000**	0.1441	0.3514	0.1913	0.1441	0.0508	0.0536
Fisher iris	0.4518	**0.5503**	0.4518	0.4518	0.2369	0.4518	0.0106	**0.5503**
Breast cancer	0.8240	0.8189	0.8240	0.8240	−0.0779	−0.0779	−0.0780	**0.8283**
Aggregation	**0.9898**	0.7338	**0.9898**	**0.9898**	0.8770	0.9866	0.6330	**0.9898**
Thyroid	0.6715	0.6715	−0.0664	**0.7339**	0.2940	−0.1332	−0.1396	0.6715
Zelnik1	0.7708	**1.0000**	0.3409	0.7852	0.7724	0.7724	**1.0000**	0.7781
Zelnik5	0.9214	**1.0000**	0.9278	**1.0000**	0.9216	0.9126	0.9839	**1.0000**
Xclara	**0.9813**	0.0001	0.0001	**0.9813**	**0.9813**	**0.9813**	0.0001	**0.9813**
Banana	**1.0000**	**1.0000**	**1.0000**	**1.0000**	**1.0000**	**1.0000**	**1.0000**	**1.0000**
Ds2c2sc13	0.3187	0.3187	0.4911	0.4911	0.5325	0.4911	0.3187	**0.5904**
2sp2glob	1.0000	**1.0000**	0.9850	0.9940	0.9985	**1.0000**	0.9970	0.9940
Cure-t1-2000n	**0.8850**	**0.8850**	**0.8850**	**0.8850**	**0.8850**	**0.8850**	**0.8850**	**0.8850**

**Table 12:** ARI values, which were obtained from the parameters, were given in Table 8 and were proposed by each index.

Dataset	Obtained ARI values for the each index							
	SI	DI	DB	CH	S_Dbw	RMSSTD	DSI	VIASCKDE
Half-kernel	0.1514	**1.0000**	0.1422	0.1421	0.1515	0.1421	**1.0000**	**1.0000**
Two spirals	0.1401	**1.0000**	0.1435	0.1401	0.1401	0.1401	0.2047	**1.0000**
Outlier	0.8463	**1.0000**	**1.0000**	0.2236	0.2322	**1.0000**	0.2271	**1.0000**
Corners	0.4581	**1.0000**	0.4581	0.4581	0.3917	0.4199	0.3330	0.3330
Cluster in cluster	0.6584	**1.0000**	0.6584	0.1365	0.1368	0.1365	**1.0000**	**1.0000**
Crescent full moon	0.2934	**1.0000**	0.2934	0.1021	0.0869	0.0955	**1.0000**	0.2341
Moon	0.3629	0.2973	0.3629	0.3629	0.3092	0.3092	**0.4916**	**0.4916**
Face	0.0646	**0.3662**	0.0747	0.0580	0.0443	0.0538	**0.3662**	**0.3662**
Wave	0.2970	**1.0000**	0.1333	0.1323	0.1323	0.1356	**1.0000**	**1.0000**
Fisher iris	0.5681	0.5681	0.5681	**0.7445**	0.2395	0.5681	0.5681	**0.7445**
Breast cancer	**0.8933**	**0.8933**	**0.8933**	**0.8933**	0.2875	0.1779	0.0669	0.2534
Aggregation	0.7975	0.0646	**0.9066**	0.4453	0.0486	0.4156	0.1149	0.0646
Thyroid	**0.6307**	0.4204	**0.6307**	**0.6307**	0.0830	0.0830	0.4204	**0.6307**
Zelnik1	0.3170	0.4352	0.3004	0.3170	0.2225	0.3007	**1.0000**	**1.0000**
Zelnik5	0.6567	0.3096	0.6567	0.3638	0.3790	0.3638	0.5003	**1.0000**
Xclara	**0.9939**	0.6270	**0.9939**	**0.9939**	0.3602	**0.9939**	0.6270	**0.9939**
Banana	0.2394	**1.0000**	0.2394	0.1369	0.1463	0.1369	1.0000	**1.0000**
Ds2c2sc13	0.3267	0.3267	0.2766	0.4531	0.0244	**0.5344**	0.0244	0.2394
2sp2glob	**0.7852**	0.5709	0.3226	0.3195	0.3185	0.3226	0.5709	**0.7852**
Cure-t1-2000n	0.6334	0.3423	0.7818	0.3303	0.1757	0.3546	0.1757	**0.8427**

**Table 13:** Obtained values for each index based on the parameters given in Table 9.

Dataset	Obtained values for the each index							
	SI	DI	DB	CH	S_Dbw	DSI	RMSSTD	VIASCKDE
Half-kernel	0.201	0.0949	1.8878	171.8984	0.5589	0.4662	0.2495	0.7125
Two spirals	0.4071	0.1317	1.1858	259.0349	0.0136	0.9957	0.28	0.8151
Outlier	0.5608	0.4291	0.4037	1075.5609	0.2099	0.9654	0.1235	0.6881
Corners	0.4614	0.2872	0.7436	2020.1068	0.0437	0.9791	0.1187	0.6268
Cluster in cluster	0.2231	0.2341	4.4083	2.5624	0.0642	0.947	0.2275	0.6052
Crescent full moon	0.2784	0.1923	1.0934	285.1423	0.0527	0.9829	0.2423	0.6623
Moon	0.2371	0.0794	1.1729	244.1722	0.3243	0.7021	0.2628	0.7002
Face	0.417	0.2217	0.9539	204.5665	0.4031	0.8557	0.2339	0.6654
Wave	0.3746	0.1291	1.1785	168.9936	0.3155	0.7862	0.2541	0.617
Fisher iris	0.6295	0.3581	0.4659	353.3674	0.4488	0.9296	0.1478	0.4722
Breast cancer	0.4306	0.1125	1.1919	493.4632	0.1958	0.9575	0.2983	0.0143
Aggregation	0.4925	0.1432	0.6452	778.9448	0.2701	0.8481	0.1497	0.6108
Thyroid	0.4359	0.0683	1.68	38.4235	0.6519	0.7913	0.1532	0.3833
Zelnik1	0.0008	0.0992	13.2535	12.7433	0.3022	0.7287	0.2171	0.541
Zelnik5	0.4663	0.2224	1.0459	413.8835	0.4593	0.7425	0.1493	0.7739
Xclara	0.6745	0.0295	1.213	7008.8746	0.0475	0.9918	0.1114	0.7814
Banana	0.3589	0.1258	1.0288	3532.2201	0.7625	0.7003	0.2146	0.82
Ds2c2sc13	0.5724	0.237	0.5829	1785.9002	0.1831	0.8928	0.1093	0.6045
2sp2glob	0.3899	0.1278	2.7973	158.408	0.6374	0.8003	0.2088	0.7146
Cure-t1-2000n	0.4514	0.1196	0.6775	1365.0774	0.3054	0.787	0.1721	0.655

**Table 14:** ARI values, which were obtained from the parameters, are given in Table 9 and were proposed by each index.

Dataset	Obtained ARI values for the each index							
	SI	DI	DB	CH	S_Dbw	DSI	RMSSTD	VIASCKDE
Half-kernel	**1.0000**	**1.0000**	0.9980	0.7901	0.7901	0.7901	**1.0000**	**1.0000**
Two spirals	0.0079	**1.0000**	0.0079	0.7524	0.0076	0.0076	0.9990	**1.0000**
Outlier	**1.0000**	**1.0000**	**1.0000**	**1.0000**	**1.0000**	**1.0000**	**1.0000**	**1.0000**
Corners	**1.0000**	**1.0000**	**1.0000**	**1.0000**	0.8261	0.8261	**1.0000**	**1.0000**
Cluster in cluster	**1.0000**	**1.0000**	0.5285	0.5360	0.5274	0.5274	**1.0000**	**1.0000**
Crescent full moon	**1.0000**	**1.0000**	0.1160	**1.0000**	0.1160	0.1160	**1.0000**	**1.0000**
Moon	0.9379	**1.0000**	0.9379	0.9379	0.2933	0.3697	0.2933	**1.0000**
Face	0.1883	0.9949	**1.0000**	0.1883	**1.0000**	**1.0000**	0.9949	**1.0000**
Wave	0.2609	**1.0000**	0.1709	0.2609	0.2528	0.2140	**1.0000**	**1.0000**
Fisher iris	0.5681	0.5681	0.5657	0.5681	0.5681	0.5638	**0.5482**	**0.5682**
Breast cancer	0.8349	**0.8522**	0.0011	**0.8522**	0.0011	0.0011	-0.0707	**0.8522**
Aggregation	0.7962	0.7338	0.7323	0.7338	**0.8154**	0.8089	0.7338	0.8089
Thyroid	0.4885	**0.5662**	0.4885	0.4885	0.4880	0.4885	-0.0255	0.4873
Zelnik1	0.9313	**1.0000**	0.3207	0.8880	**1.0000**	0.9680	0.9771	**1.0000**
Zelnik5	**1.0000**	**1.0000**	**1.0000**	**1.0000**	**1.0000**	**1.0000**	**1.0000**	**1.0000**
Xclara	0.9861	**0.9904**	0.3936	0.9880	0.3936	0.3936	0.3936	**0.9904**
Banana	**1.0000**	**1.0000**	0.8308	**1.0000**	**1.0000**	0.8278	**1.0000**	**1.0000**
Ds2c2sc13	0.3187	0.3187	0.3180	0.3187	**0.7624**	0.3187	0.3165	0.4260
2sp2glob	**1.0000**	**1.0000**	**1.0000**	**1.0000**	**1.0000**	**1.0000**	**1.0000**	**1.0000**
Cure-t1-2000n	**0.8850**	**0.8850**	**0.8850**	**0.8850**	**0.8850**	**0.8850**	**0.8850**	**0.8850**

**Table 15:** The number of highest ARI values that each index reached.

Index	# of datasets that each index was the best on the different algorithms			
	DBSCAN	Spectral Clustering	HDBSCAN	Total
SI	*11*	*4*	*9*	*24*
DI	*13*	*10*	*16*	*39*
DB	*7*	*5*	*6*	*18*
CH	*13*	*4*	*8*	*25*
S_Dbw	*5*	*0*	*9*	*14*
DSI	*8*	*7*	*5*	*20*
RMSSTD	*5*	*3*	*11*	*19*
VIASCKDE (proposed index)	***15***	***15***	***17***	***47***

## Data Availability

Python implementation of the proposed index is shared on GitHub (https://github.com/senolali/VIASCKDE).
